# Improving Delegation of New Patient Referral Allocations to Manage the Workload Burden of Staff

**DOI:** 10.1192/bjo.2025.10522

**Published:** 2025-06-20

**Authors:** Siyament Sacaklidir, Asifa Shabbir, Brunda Chandra

**Affiliations:** Surrey and Borders Partnership NHS Foundation Trust, Guildford, United Kingdom

## Abstract

**Aims:** Referrals to Old Age Community Mental Health Team (CMHT OP) Guildford/Waverley have increased in number and workload. The purpose was to see the number of referrals received and how to improve the referral allocation practice concerning delegation to manage the workload burden on staff.

**Methods:** 1230 referrals were reviewed. The quantitative data was primarily taken from allocation meeting documentation. Additionally, information was gathered from senior staff and relevant documentation of allocation meetings on the electronic patient records when there was vague information.

**Results:** The data shows that during the first cycle from April to September 2023, CMHT OP Guildford and Waverley received 579 referrals. 90% (521) of the referrals were accepted, and 10% (58) were declined due to inappropriate referrals. There were 412 routine, 65 sooner and 92 urgent referrals received. Senior team members assessed and stepped down 21 of the urgent referrals. 459 patients were referred due to organic conditions, 76 patients were referred due to functional conditions, and 23 patients were referred for a mix of functional and organic conditions. 85 patients were transferred to the Care Home Pathway (CHP) service, 18 patients were transferred to the Young Onset Dementia (YOD) service, and 25 patients were transferred to the Integrated Care Team (ICT). The first cycle of the audit was presented to the team, and steps of interventions were agreed upon for the second cycle. The re-audit data shows that from October 2023 to March 2024, there were 651 referrals. 88% of the referrals were accepted, and 12% were declined due to inappropriate referrals. There were 510 routine, 57 sooner and 84 urgent referrals received. Senior team members stepped down 26 of the urgent referrals. 523 patients were referred due to organic conditions, 88 patients were referred due to functional conditions, and 48 patients were referred for a mix of functional and organic conditions. 76 patients were transferred to the CHP service, 15 patients were transferred to the YOD service, and 51 patients were transferred to the ICT.

**Conclusion:** The audit data objectively reflects an increasing trend in referrals between the first and second cycles. Intervention after the first audit cycle showed increased use of advice and guidance services for declined referrals, increased step-down of urgent referrals and an increased number of patients delegated to other services, particularly the integrated care team, which shows a more confident referral allocation process.

